# Novel vaccine potential of Rv3131, a DosR regulon-encoded putative nitroreductase, against hyper-virulent *Mycobacterium tuberculosis* strain K

**DOI:** 10.1038/srep44151

**Published:** 2017-03-08

**Authors:** Kee Woong Kwon, Woo Sik Kim, Hongmin Kim, Seung Jung Han, Mi-Young Hahn, Jong Seok Lee, Ki Taek Nam, Sang-Nae Cho, Sung Jae Shin

**Affiliations:** 1 Department of Microbiology, Yonsei University College of Medicine, Seoul 03722, South Korea; 2 Institute for Immunology and Immunological Diseases, Brain Korea 21 PLUS Project for Medical Science, Yonsei University College of Medicine, Seoul 03722, South Korea; 3 Department of Microbiology Research, International Tuberculosis Research Center, Changwon 51755, South Korea; 4 Severance Biomedical Science Institute, Brain Korea 21 PLUS Project for Medical Science, Yonsei University College of Medicine, Seoul 03722, South Korea

## Abstract

Accumulating evidence indicates that latency-associated *Mycobacterium tuberculosis* (Mtb)-specific antigens from the dormancy survival regulator regulon (DosR) may be promising novel vaccine target antigens for the development of an improved tuberculosis vaccine. After transcriptional profiling of DosR-related genes in the hyper-virulent Beijing Mtb strain K and the reference Mtb strain H37Rv, we selected Rv3131, a hypothetical nitroreductase, as a vaccine antigen and evaluated its vaccine efficacy against Mtb K. Mtb K exhibited stable and constitutive up-regulation of *rv3131* relative to Mtb H37Rv under three different growth conditions (at least 2-fold induction) including exponential growth in normal culture conditions, hypoxia, and inside macrophages. Mice immunised with Rv3131 formulated in GLA-SE, a well-defined TLR4 adjuvant, displayed enhanced Rv3131-specific IFN-γ and serum IgG2c responses along with effector/memory T cell expansion and remarkable generation of Rv3131-specific multifunctional CD4^+^ T cells co-producing TNF-α, IFN-γ and IL-2 in both spleen and lung. Following challenge with Mtb K, the Rv3131/GLA-SE-immunised group exhibited a significant reduction in bacterial number and less extensive lung inflammation accompanied by the obvious persistence of Rv3131-specific multifunctional CD4^+^ T cells. These results suggest that Rv3131 could be an excellent candidate for potential use in a multi-antigenic Mtb subunit vaccine, especially against Mtb Beijing strains.

Tuberculosis (TB) caused by *Mycobacterium tuberculosis* (Mtb) is a major health threat worldwide^1^. With the increasing prevalence of HIV infection and multiple drug-resistant strains, TB remains one of the leading infectious diseases responsible for high morbidity and mortality in the past decades^1^,^2^,^3^. Moreover, according to the World Health Organization (WHO), nearly one-third of the world’s population is estimated to be latently infected with Mtb, approximately 5–10% of whom will progress to active disease during their lifetime through reactivation^4^. This reservoir of latent bacteria is responsible for disease transmission, which impedes attempts to control TB^5^,^6^,^7^,^8^. The TB vaccines currently in clinical trials are mostly designed as prophylactics. Essentially, these prophylactic vaccines target antigens (Ags) that are expressed during the early phase of infection; however, these early Ags are markedly down-regulated in the late phase when the bacteria are in the dormant state^6^. The Bacillus Calmette-Guérin (BCG) vaccine, which has been in use since 1921^9^, protects children from severe TB but exhibits unreliable protective efficacy against adult pulmonary TB^10^,^11^,^12^. Thus, this fundamental imperfection of the vaccines that are currently available or under development renders them inappropriate for conferring long-lasting protection against TB and highlights the importance of discovering and selecting new Mtb vaccine target Ags.

The phenotypic, genotypic and pathogenic variation among Mtb strains should also be taken into account, as these factors can contribute to vaccine efficacy^13^,^14^, and different Mtb strains exhibit different levels of virulence^13^. Importantly, the use of laboratory-adapted Mtb strains such as H37Rv or Erdman to evaluate vaccine efficacy limits current vaccine studies^15^,^16^. Notably, one of the putative reasons for MVA85 vaccine failure in BCG-vaccinated infants^17^ might be the underestimation of Mtb genetic diversity and virulence^15^. Specifically, one family known as the Beijing genotype has drawn considerable attention over the past decade, as Mtb strains in this family have been associated with major TB outbreaks worldwide, and this strain is highly dominant in East Asia^13^,^18^,^19^. Moreover, epidemiological studies have suggested that extensive BCG vaccination may have functioned as a selective factor for the emergence of the Beijing genotype^13^,^14^. Most TB patients experience latent infection before the onset of active disease. Thus, latency-associated Ags that are immunologically detected and specifically expressed during this phase are of particular interest as potential candidates in TB vaccine development^20^. One characteristic of Beijing strains is that this genotype is significantly associated with increased risk for relapse^21^,^22^,^23^; this high relapse rate might be related to the high expression of latency-associated genes, including the dormancy survival regulator (DosR) based on mice model study^18^,^24^. This DosR regulon, which is collectively transcribed by the DosR transcription factor, consists of ~50 genes (1.3% of the Mtb genome) classified into 9 functional categories^25^ and plays an important role in Mtb adaptation in adverse conditions^7^. Given the ability of Mtb Beijing strains to constitutively overexpress DosR regulon-related genes and their immunodominant nature^6^,^26^, DosR regulon-encoded Ags have been considered as potential candidates for the development of improved TB vaccines^27^. During exponential growth *in vitro* and intracellular infection of macrophages, *rv3131* is the most up-regulated of the DosR regulon-related genes in Korean Beijing Mtb strain K, the predominant strain in South Korea, relative to Mtb H37Rv; therefore, we selected Rv3131 as a vaccine candidate in the present study. Rv3131 is a hypothetical nitroreductase that includes two DosR binding sites that are essential and uniquely conserved in Mtb^28^. Similar to other nitroreductases such as Rv2032 and Rv3127, Rv3131 is speculated to play a protective role against nitrogen stress by detoxifying nitrogen-containing by-products inside the host^29^,^30^. In addition, one recent report indicated that Rv3131 exhibits a strong immunogenic effect, which is mediated by activation of the TLR2 pathway^7^. In addition, *rv3131* was significantly up-regulated (up to 40-fold) under *in vitro* dormancy conditions in Mtb clinical isolate 1254^8^. Nevertheless, Rv3131 is the least characterised DosR-encoded latency Ag in TB vaccine testing in pre-clinical animal models. Thus, this Ag is a potential vaccine candidate due to its overexpression in Mtb Beijing strains and its immunogenicity.

In this study, we investigated the immunogenicity and protective efficacy of Rv3131 formulated in GLA-SE (glucopyranosyl lipid adjuvant-stable emulsion), a well-defined TLR4 adjuvant, as a subunit vaccine platform against hyper-virulent Mtb K challenge.

## Results

### The *rv3131* transcript is up-regulated in the exponential growth phase, hypoxic condition and inside macrophages, regardless of growth phase, in virulent Mtb isolate

**Figure 1:**
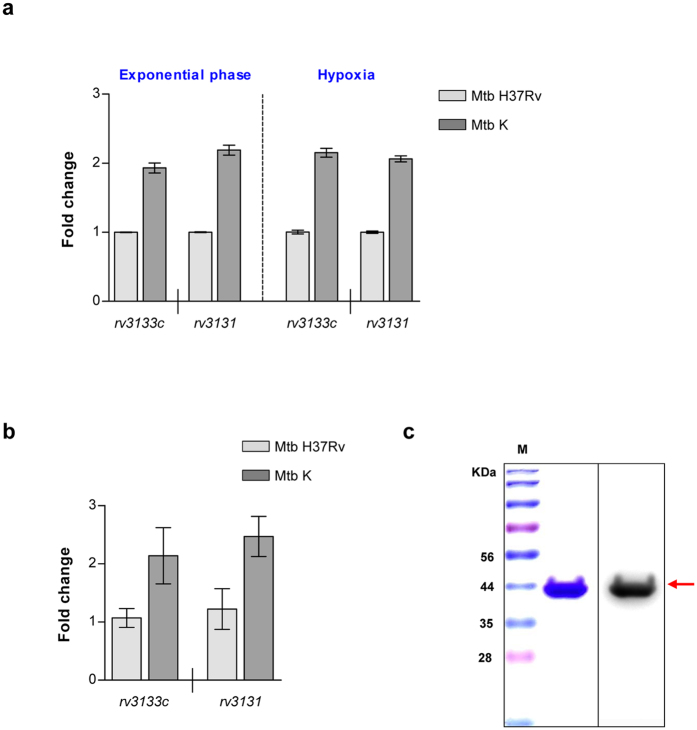
r*v3131* is stably up-regulated in virulent Korean Beijing *Mycobacterium tuberculosis* strain K. (**a**) The expression level of *rv3131* was evaluated using a qRT-PCR. The differential fold changes between Mtb H37Rv (light grey bar) and Mtb K (dark grey bar) under different growth conditions, exponential phase and hypoxic-culture condition, were determined using the ΔΔCt method, and 16 S rRNA expression was used as an endogenous control. *rv3133c (dosR*) was used as a positive control. (**b**) The qRT-PCR was further used for *rv3131* expression of Mtb inside BMDMs. The differential fold changes between Mtb H37Rv (light grey bar) and Mtb K (dark grey bar) were determined using the ΔΔCt method, and 16 S rRNA expression was used as an endogenous control. *rv3133c (dosR*) was used as a positive control. (**c**) Coomassie blue-stained 10% SDS-PAGE with recombinant Rv3131 protein (left panel) purified under endotoxin-free conditions and confirmed by Western blot with an anti-histidine antibody (right panel). M: Molecular weight standard marker.

To clarify the potential of Rv3131 as a vaccine Ag candidate against hyper-virulent Beijing Mtb isolates, we first performed a microarray analysis to compare gene expression between the Mtb K and H37Rv strains growing exponentially and under hypoxic condition in 7H9 media. Whole genome microarray analysis indicated that DosR regulon-associated genes were highly up-regulated in Mtb K relative to H37Rv under normal *in vitr*o culture conditions. Among these up-regulated DosR regulon-related genes, *rv3131* showed the greatest increase, with an approximately 5.6-fold induction relative to Mtb H37Rv (Supplementary Table S1). Under hypoxic conditions, *rv3131* was also up-regulated about 2.7-fold relative to Mtb H37Rv (Supplementary Table S1). The expression level of *rv3131* was further confirmed using a quantitative real-time PCR (qRT-PCR). Along with microarray results, the stable and constitutive up-regulation of *rv3131* (at least 2-fold induction compared to that in Mtb H37Rv) was confirmed in Mtb K under both growth conditions (Fig. 1a). Furthermore, bone marrow-derived macrophages (BMDMs) were infected with each Mtb strain at a multiplicity of infection (MOI) of 10 for 24 h to effectively mimic the harsh conditions *in vitro*, and *rv3131* expression was evaluated using a qRT-PCR. Relative to H37Rv, hyper-virulent Mtb K showed marked *rv3131* expression while the bacteria persisted inside BMDMs (Fig. 1b). This up-regulation of *rv3131* in Mtb K in three culture conditions supports the proposal of Rv3131 as a possible vaccine candidate Ag for virulent Beijing Mtb isolates. Next, recombinant Rv3131 protein was extracted via cell sonication and then purified using cobalt resin under endotoxin-free conditions. To remove residual endotoxin, purified Rv3131 was treated with polymyxin B agarose. The purity and expected ~38 kDa molecular weight of Rv3131 were confirmed by sodium dodecyl sulfate-polyacrylamide gel electrophoresis (SDS-PAGE) and Western blotting (Fig. 1c).

### Immunogenicity and immunological memory induced by Rv3131 immunisation

**Figure 2:**
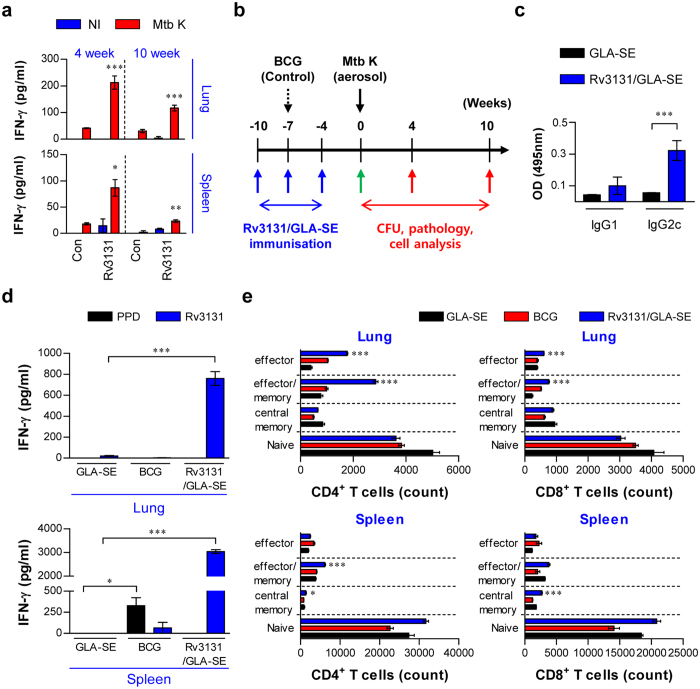
*Ex vivo* stimulation with Rv3131 elicits an Ag-specific IFN-γ response in both lung and spleen cells from *Mycobacterium tuberculosis* K-infected mice; immunisation with Rv3131 induces a Th1-type T cell response. (**a**) The Rv3131-specific IFN-γ response was analysed after stimulating lung and spleen cells from each mouse with Rv3131 at 4 or 10 weeks after Mtb K infection via aerosolisation. The graphs illustrate the mean ± SD of 3 samples from each of 3 mice. One representative plot from three independent experiments is shown. *p <* *0.05, **p <* *0.01, and ***p <* *0.001 compared to the non-stimulated infection group (Con). Rv3131: Rv3131-stimulated cells; NI: not infected. (**b**) Scheme of the experimental design for Rv3131 subunit vaccine testing. Mice (*n* = 17 per group) were immunised with Rv3131/GLA-SE or GLA-SE with only three intramuscular injections (blue arrows) before Mtb K aerosol challenge (black arrow). BCG was injected subcutaneously at the time of the 2^nd^ Rv3131/GLA-SE injection (dashed arrow). Immunological analysis was performed before (green arrow; **c**–**e**) and after Mtb infection (red arrow). Bacterial counts and histopathological analysis in each group were determined at the indicated time points after Mtb infection (red arrow). (**c**) Serum antibody response at four weeks after the final immunisation with Rv3131/GLA-SE or adjuvant alone. The Rv3131-specific IgG1 and IgG2c responses in each group were measured in serum samples. (**d**) The level of IFN-γ secreted by lung and spleen cells from each fully immunised group in response to Rv3131 (5 μg/mL) or PPD (2 μg/mL) stimulation was detected by ELISA. *p <* *0.05 and ***p <* *0.001 compared to GLA-SE-immunised mice. (**e**) Analysis of T cell subpopulations after the final immunisation. The number of infiltrating CD4^+^, CD8^+^ effector (CD44^hi^CD62L^−^CD127^−^), effector/memory (CD44^hi^CD62L^−^CD127^+^), central memory (CD44^hi^CD62L^+^CD127^+^), and naïve (CD44^lo^CD62L^+^CD127^+^) T cells in the lungs and spleens of each immunised group were analysed by flow cytometry. The data are presented as the mean ± SD from 5 mice in each group. The significance of the differences was determined using an unpaired *t*-test. *p <* *0.05, **p <* *0.01, and ***p <* *0.001 compared to the GLA-SE-alone group.

Prior to testing Rv3131 vaccine efficacy, we investigated whether Rv3131 recognition by the host immune system induces an immunological memory response during *in vivo* infection. Thus, the ability to generate an Ag-specific IFN-γ response upon stimulation with Rv3131 was examined in lung and spleen cells from Mtb K-infected mice at early (4 weeks) and late (10 weeks) time points post-infection. The lung and spleen cells of Mtb K-infected mice produced IFN-γ in response to Rv3131, indicating that Rv3131 is recognised during the course of Mtb K infection (Fig. 2a). Next, the type of Rv3131 vaccine-induced immune response and memory T cell expansion were investigated at 4 weeks after the final immunisation (Fig. 2b, green arrow). To identify whether Th1-type T cell responses were achieved through Rv3131/GLA-SE immunisation, Rv3131-specific antibody titres were analysed. The Rv3131/GLA-SE-immunised group exhibited an elevated Rv3131-specific IgG2c response but not an IgG1 response (Fig. 2c). Furthermore, we analysed the production of Ag-specific IFN-γ, IL-4, and IL-5, and the type of memory T cell subpopulations generated in the spleen and lung following Rv3131 immunisation. The production of IFN-γ, but not the Th2-related cytokines IL-4 and IL-5 (Supplementary Fig. S1), was significantly higher in lung and spleen cells from mice immunised with Rv3131 compared to those from the GLA-SE only group, while an elevated IFN-γ response to PPD (purified protein derivative) was observed only in the spleen cells of BCG-immunised mice (Fig. 2d). Next, the frequencies at which effector/memory T cell subpopulations infiltrated the lung and spleen after the final immunisation were analysed by flow cytometric analysis using the gating strategy depicted in Supplementary Fig. S2. The proportion of CD4^+^/CD8^+^ effector/memory and effector T cells in the lungs was significantly greater in the Rv3131/GLA-SE-immunised group than in the GLA-SE only-immunised group (Fig. 2e, top panel). In addition, the proportion of central memory CD4^+^/CD8^+^ T cells was significantly increased only in spleen cells from the Rv3131/GLA-SE-immunised group compared to the GLA-SE-immunised group. Additionally, the proportion of effector/memory T cells increased only in the CD4^+^ T cell population from the spleens of the Rv3131/GLA-SE-immunised group (Fig. 2e, bottom panel). Importantly, robust induction of effector/memory CD4^+^ T cells was observed in both lung and spleen from the Rv3131/GLA-SE-immunised group. These results indicate that Rv3131 has immunogenic potential as a subunit vaccine Ag due to its generation of Ag-specific effector/memory CD4^+^ T cells and a Th1-polarized response.

### Evaluation of Ag-specific multifunctional T cell generation following Rv3131-immunisation prior to Mtb K challenge

**Figure 3:**
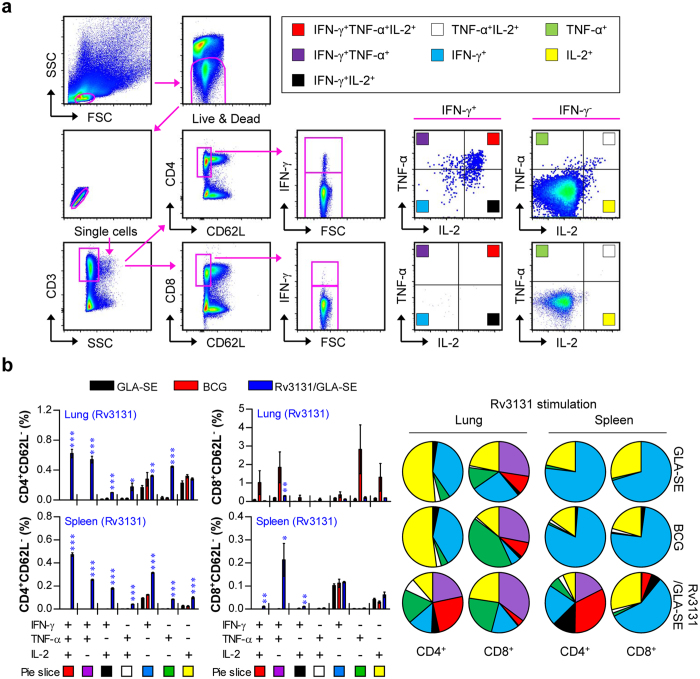
Induction of Ag-specific multifunctional T cells in the spleens and lungs in Rv3131-immunised mice. Each group of mice was immunised and euthanised as described in the Methods section. Four weeks after the final immunisation, the mice in each group were sacrificed, and their lung and spleen cells were treated with Rv3131 (5 μg/mL) at 37 °C for 12 h in the presence of Golgi Stop. (**a**) The gating strategy used to identify Ag-specific multifunctional T cell populations. (**b**) Upon stimulation with the Rv3131 vaccine Ag, the percentages of Ag-specific, multifunctional CD4^+^CD62L^−^ and CD8^+^CD62L^−^ T cells producing TNF-α, IFN-γ and/or IL-2 in the lung and spleen cells from each immunised group were evaluated using flow cytometry. The mean frequencies of cells producing effector cytokines are shown as pie charts. The results are expressed as the mean ± SD for 5 mice from each group. The significance of differences was determined using an unpaired *t*-test. A *p* value* *<* *0.05 was considered statistically significant. *p <* *0.05, **p <* *0.01, and ***p <* *0.001 compared to the GLA-SE alone group.

Although a consensus on protective correlates for TB vaccine development has not been reached in humans, recent studies have suggested the protective contribution of multifunctional T cells and a Th1-mediated immune response against Mtb infection in animal models^31^,^32^,^33^. Thus, we next assessed the frequency at which Rv3131-specific IFN-γ-, TNF-α- and IL-2-producing bi-functional or multifunctional CD4^+^/CD8^+^ T cells were generated upon *ex vivo* re-stimulation with Rv3131 after the final immunisation. After stimulation with Rv3131, CD4^+^ and CD8^+^ T cells from the lung and spleen were stained for intracellular cytokines, and the phenotype of the responding T cells was then analysed by multi-colour flow cytometry (Fig. 3a). Based on the ability to release effector cytokines (TNF-α, IFN-γ and IL-2), the Ag-specific memory phenotype of CD4^+^/CD8^+^ T cells was characterised at the single-cell level. The CD4^+^/CD8^+^ T cells within the CD62L-negative population were analysed, and the CD4^+^CD62L^−^ and CD8^+^CD62L^−^ T cell populations were divided into seven subpopulations according to their production of TNF-α, IFN-γ and/or IL-2 in any combination. Upon stimulation with Rv3131, the Rv3131/GLA-SE-immunised mice displayed significantly greater levels of bi-functional (double-positive) or multifunctional (triple-positive) CD4^+^ T cells (co-producing IFN-γ, TNF-α and/or IL-2) in the lung and spleen, while a relatively high level of multifunctional CD8^+^ T cells was observed only in spleens from Rv3131/GLA-SE-immunised mice relative to those of the control group, indicating that Rv3131/GLA-SE preferentially induced Ag-specific, multifunctional CD4^+^ T cell responses in both the lung and spleen (Fig. 3b).

### Protective efficacy of the Rv3131 subunit vaccine against the hyper-virulent Beijing Mtb K infection in a prophylactic setting

**Figure 4:**
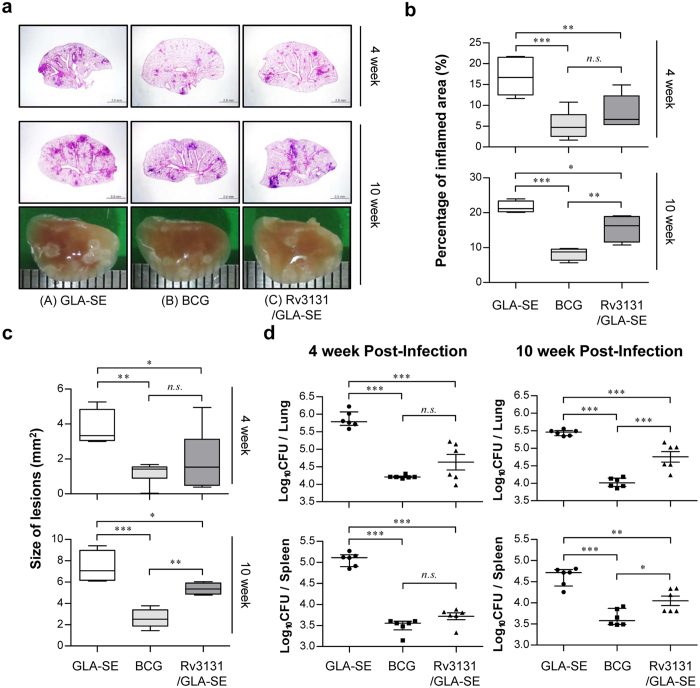
The efficacy of the Rv3131 subunit vaccine as a prophylactic against hyper-virulent *Mycobacterium tuberculosis* K infection was evaluated with histopathological analysis and bacterial CFUs. Mice were infected with Mtb K strain via aerosolisation, and lungs and spleens were removed at 4 weeks and 10 weeks post-infection for subsequent analysis. (**a**, top and middle panel) The superior lobe of the right lung from each immunised mouse stained with H&E at 4 or 10 weeks after Mtb K challenge; (**a**, bottom panel) representative gross lung pathology at 10 weeks post-infection. (**b**) The data represent the percentages of the superior lobe of the right lung showing inflammation and are shown as box-and-whisker plots showing the minimum and maximum values (6 mice per group at each designated time point). (**c**) The size of the lesions was analysed and is depicted as box-and-whisker plots showing the minimum and maximum values (6 mice per group at each designated time point). (**d**) The CFU in the lungs and spleens of each group at 4 and 10 weeks post-infection was analysed by counting the bacteria. The data are expressed as the median with IQR log_10_CFU/organ (6 mice per group at each designated time point). One-way ANOVA followed by Tukey’s multiple comparison test was used to evaluate significance. *p <* *0.05, **p <* *0.01, and ***p <* *0.001.

Based on the immunological potential of Rv3131 as a potent Mtb vaccine target Ag, we next evaluated the protective efficacy of Rv3131 immunisation against Mtb K challenge. Four weeks after the last immunisation, the mice were aerogenically challenged with highly virulent Mtb K. Four and ten weeks after Mtb K infection, histopathological analysis and bacterial burden were examined in the GLA-SE-, BCG- and Rv3131/GLA-SE-immunised groups. Haematoxylin and eosin (H&E) staining of lung sections at 4 and 10 weeks post-challenge and the gross lung pathology of Mtb K-infected mice at 10 weeks post-challenge indicated improved lung inflammation (4 weeks, *p* < 0.01; 10 weeks, *p* < 0.05) and reduced lesion size (4 weeks, *p* < 0.05; 10 weeks, *p* < 0.05) in the Rv3131/GLA-SE-immunised group relative to the GLA-SE-control group (Fig. 4a,b and c). In addition, the bacterial burden in both the lung and the spleen was significantly lower in the Rv3131/GLA-SE-immunised group than the GLA-SE-control group at 4 weeks post-challenge (lung, *p* < 0.001; spleen, *p* < 0.001). Furthermore, the protection achieved by Rv3131/GLA-SE immunisation was maintained at 10 weeks post-infection relative to the protection in the GLA-SE-control group (lung, *p* < 0.001; spleen, *p* < 0.01), although protection decreased somewhat relative to that in the BCG-immunised group (lung, *p* < 0.001; spleen, *p* < 0.05) (Fig. 4d). These results suggest that Rv3131 subunit vaccination may offer protective efficacy similar to that observed in BCG against Mtb Beijing strains, particularly when administered as a single Ag at early time points post Mtb K infection.

### Maintenance of long-lived Rv3131-induced Th1-type multifunctional T cell immunity after challenge with virulent Mtb K

**Figure 5:**
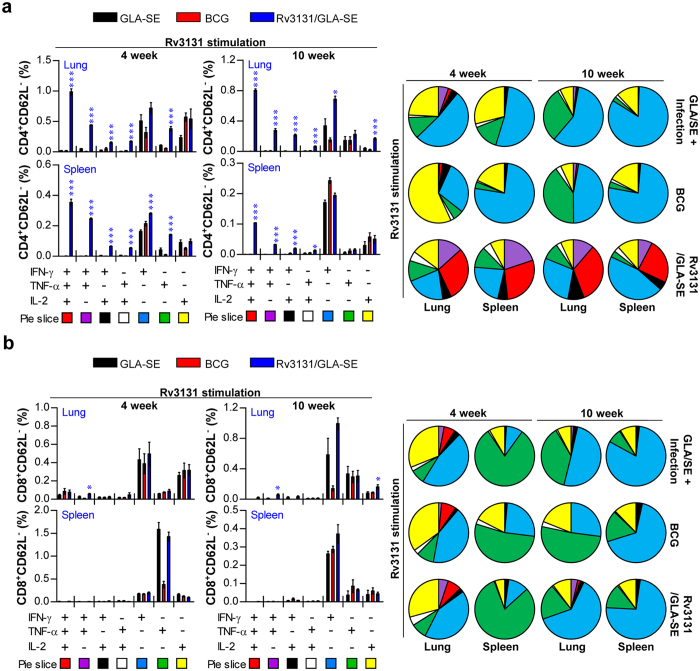
Analysis of Rv3131-specific multifunctional T cells after challenge with *Mycobacterium tuberculosis* K. The mice in each group were sacrificed at 4 or 10 weeks post-infection, and their lung and spleen cells were stimulated with Rv3131 (5 μg/mL) at 37 °C for 12 h in the presence of GolgiStop. Upon stimulation, the percentages of Ag-specific, multifunctional CD4^+^CD62L^−^ (**a**) and CD8^+^CD62L^−^ (**b**) T cells producing TNF-α, IFN-γ and/or IL-2 in each immunised group were evaluated using flow cytometry. The mean frequencies of cells producing effector cytokines are shown as pie charts. The results are expressed as the mean ± SD for 6 mice in each group. The significance of the differences was determined using an unpaired *t*-test. A *p* value* *<* *0.05 was considered statistically significant. *p <* *0.05, **p <* *0.01, and ***p <* *0.001 compared to the GLA-SE alone group.

To examine whether the enhanced pulmonary protection induced by the Rv3131 subunit vaccine correlated with the generation of Rv3131-specific CD4^+^ or CD8^+^ T cells expressing effector cytokines, we evaluated the frequency of multifunctional T cells after *in vitro* stimulation with PPD (Supplementary Fig. S3) or Rv3131 (Fig. 5) at 4 and 10 weeks post-infection. While the levels of double-positive (IFN-γ^+^TNF-α^+^, TNF-α^+^IL-2^+^ and IFN-γ^+^IL-2^+^) and triple-positive (IFN-γ^+^TNF-α^+^IL-2^+^) CD4^+^ T cells in the lung cells of immunised mice did not differ significantly upon PPD stimulation (Supplementary Fig. S2a), stimulation with Rv3131 at 4 and 10 weeks post-infection resulted in an Ag-specific increase in the levels of double-positive and triple-positive CD4^+^ T cells in the Rv3131/GLA-SE-immunised group relative to the GLA-SE-immunised group (Fig. 5a). PPD stimulation induced multifunctional T cells only in BCG-immunised mice (Supplementary Fig. S2). Hence, the Rv3131/GLA-SE-immunised group showed an increased frequency of Rv3131-specific multifunctional CD4^+^ T cells in the spleen relative to the GLA-SE-immunised group in response to Rv3131 (Fig. 5a), but the same did not hold true for CD8^+^ T cells in either the lung or the spleen (Fig. 5b). These results indicate that Rv3131/GLA-SE immunisation induces a sustained level of multifunctional CD4^+^ T cells, resulting in lasting protection against the hyper-virulent Mtb K isolate.

## Discussion

Although the first critical step in developing an improved TB vaccine requires the discovery and selection of vaccine target Ags with proven immunogenicity and protective efficacy in preclinical animal testing, only a few early-stage Ags, such as Ag85A, Ag85B, and ESAT-6, are considered promising Ags for prophylactic vaccines^17^,^34^,^35^. However, there is accumulating evidence that the inclusion of latency-associated Ags, specifically, Ags encoded by the DosR regulon, will be important in the development of a more potent TB vaccine^6^. For example, H56 (developed by the Statens Serum Institut, Demark) and ID93 (developed by the Infectious Disease Research Institute, USA), the most advanced multi-stage subunit vaccines in clinical trials, contain the latency-associated Ags Rv2660c and Rv1813, respectively^36^,^37^.

In addition, extensive genetic variation may lead to altered vaccine efficacy and gene expression pathways important for TB vaccine development. For example, through mathematical modelling analyses, Cohen *et al*. predicted that incomplete understanding of mycobacterial strain diversity could have considerable negative effects on vaccine efficacy due to strain replacement by variants of Mtb not targeted by the vaccine^38^. Moreover, Homolka *et al*. suggested that the genetic diversity in Mtb strains reinforces the need to embrace the challenge of using diverse Mtb strains as a common step in vaccine testing and drug screening^39^.

Thus, we hypothesised that the clinically predominant Mtb genotype- and strain-specifically overexpressed Ags may be good vaccine target Ags. Through microarray analysis of gene transcripts, we finally identified Rv3131 as fulfilling the above standard among DosR regulon-encoded genes. To the best of our knowledge, this is the first time that the DosR regulon-related Rv3131 was shown to be effective against challenge with a highly virulent Beijing Mtb clinical strain.

Importantly, the gene expression profile in the hyper-virulent Mtb Beijing strain is obviously different from that of the laboratory-adapted Mtb H37Rv strain, and many genes (*dosR, rv3130c, hspX* and *fdxA*) that comprise the DosR regulon have been reported to be constitutively and specifically overexpressed in Mtb Beijing clinical isolates^40^,^41^,^42^,^43^. These genes were also relatively up-regulated in the Mtb strain K relative to Mtb H37Rv, indicating that our results are in good agreement with data from previous studies (Fig. 1a and Supplementary Table S1). Surprisingly, the basal transcription level of DosR genes in the Beijing family was highly up-regulated (up to 40- to 50-fold) relative to that in the non-Beijing family in the early log phase of growth in *in vitro* cultures, suggesting that the Beijing family was pre-adapted or prepared for future environmental stress^39^,^40^. In fact, in addition to the constitutive overexpression of Rv3131, basal Rv3131 expression is required because it is an essential factor for the survival and growth of Mtb^28^, indicating that Rv3131 may be a good candidate vaccine Ag if it induces the appropriate anti-Mtb Th1 immune response.

In this study, we investigated whether Rv3131 could induce an Ag-specific IFN-γ response during Mtb K infection (Fig. 2a) and the type of T cell response induced by vaccination with Rv3131 and GLA-SE (Figs 2, 3, and 5) pre- and post-challenge. Our study showed that Rv3131 induced an Ag-specific IFN-γ response during Mtb K infection and that vaccination with Rv3131 and GLA-SE induced a strong Ag-specific memory T cell response. Furthermore, the Rv3131 subunit vaccine induced a robust and sustained Th1-based memory response (Figs 2, 3 and 5) and comparable protective efficacy against Mtb K, in contrast to BCG-induced protection as a single Ag subunit vaccination at 4 weeks post-infection (Fig. 4).

Recently, Zvi *et al*. reported that 189 genes from the 3,989 ORF products of the Mtb genome were selected as putative vaccine candidates *in silico* by a genome-scale dataset constructed by comprehensive data mining and bioinformatics^44^. Among the 189 vaccine candidates, Rv3131 and Rv3127, another DosR regulon-encoded nitroreductase and the second-highest ranked gene in our transcriptional profiling study, were included on the list of the 45 top hits. In addition to the theoretical analysis, experimental evidence indicates that Rv3131 is a T cell Ag. For example, Rv3131-elicited, Mtb-specific T cell immune responses were reported in tuberculin skin test-positive subjects but not in healthy controls or TB patients^45^. As another example, Rv3131 exhibited the most immunogenic Ag potential relative to the other DosR-related Ags tested in terms of its ability to induce IFN-γ in a latently infected Gambian population^46^.

Although IFN-γ is required to control Mtb infection, recent publications have revealed that the IFN-γ response alone is not a sufficient correlate of protective efficacy^47^,^48^. In addition, numerous studies have recently emphasised that both the magnitude and the quality of the T cell response appear to be significant factors in establishing protective memory^31^,^32^,^47^,^48^. Furthermore, the induction of multifunctional T cells that co-express IFN-γ, TNF-α and IL-2 has been shown to provide a good correlate of protection against a number of infectious diseases, including *Leishmania major* and Mtb^31^,^32^,^33^,^49^,^50^. For these reasons, the paradigm shift in vaccine-induced protection has led us to concentrate on Ag-specific CD4^+^ T cells with multifunctional properties in the lungs. After Rv3131/GLA-SE immunisation, we found a significant increase in the number of Ag-specific CD4^+^ T cells producing either three or two effector cytokines after *in vitro* stimulation with Rv3131 in lung and spleen cells (Fig. 3b), indicating the existence of ready-to-be expanded multifunctional CD4^+^ T cells and that their Ag-specific expansion is vital for vaccine development following the recognition of Mtb infection.

Based on these results, we evaluated the protective effect of Rv3131/GLA-SE vaccination against the Mtb K strain in a murine model. We showed that Rv3131/GLA-SE-immunised mice displayed improved lung pathology and reduced bacterial growth similar to that observed in BCG-immunised mice, particularly at 4 weeks post-infection (Fig. 4). However, the protective efficacy observed in the Rv3131/GLA-SE immunised-group at 10 weeks post-infection was moderate compared to that observed at 4 weeks in terms of bacterial reduction and the size of inflamed lesions (Fig. 4). We suspect that the reduced protective efficacy in the Rv3131/GLA-SE-immunised group may be due to two possible reasons: (1) the reduced Th1-biased response at 10 weeks post-infection relative to that at 4 weeks post-infection (Figs 2a and 5) and (2) the differential expression of Rv3131 at 4 weeks and 10 weeks post-infection. Our group previously reported that Th1 immunity decreased rapidly and that the CD4^+^CD25^+^Foxp3^+^ regulatory T cell (Treg) population increased after 4 weeks post-challenge with hypervirulent Mtb Beijing strain K, whereas challenge with the laboratory strain H37Rv did not cause these changes^51^. Thus, the elevated level of Tregs 4 weeks post-infection may reduce the Rv3131/GLA-SE immunisation-conferred, Th1-biased response at 10 weeks. In addition, the time at which Rv3131 is produced by Mtb may differ according to infection stage. In regard to human Mtb infection, it is generally believed that Mtb is latent for at least the first 2–3 years post-infection and later manifests as active TB with disease progression^4^,^5^. In contrast, Mtb are actively growing in the lungs by 4 weeks post-infection and a steady-state chronic infection is established when strong T cell responses are initiated at 3–4 weeks post-infection in a mouse model, particularly a C57BL/6 model. As *rv3131* was among the DosR regulon-encoded genes expressed at a higher level during the exponential growth phase under normal culture conditions (Fig. 1 and Supplementary Table S1), and Mtb are actively growing in organs during the early stage of lung Mtb infection in mice, Rv3131 expression may be higher during this stage (4 weeks post-infection) than at later time points (10 weeks post-infection). Nevertheless, Rv3131/GLA-SE immunisation induced multifunctional CD4^+^ T cells in the lung and spleen at 4 and 10 weeks post Mtb K infection (Fig. 5), suggesting that the induction and maintenance of multifunctional, Rv3131-specific T cells during Mtb infection seems to play an important role in conferring protection against the highly virulent Mtb strain K.

Although subunit protein vaccines are a promising strategy for preventing TB and boosting the BCG vaccine due to the specificity of immunogenic Ags and safety^52^, the limited choice of Ag in subunit vaccines reduces their ability to stimulate a complete protective immune response. In addition, immune responses to latency-associated TB Ags have been related to control of latent Mtb infection, and lack of immune response to latency-associated Ags following BCG vaccination has been reported^53^. Hence, latency-associated, DosR regulon-encoded Ags have been largely uncharacterised in TB vaccine development. Moreover, one of major drawbacks in TB vaccine development is that the expression of early Ags is significantly down-regulated in the late stage of Mtb infection^36^. Interestingly, BCG also possesses a functional DosR regulon^54^ and up-regulation of an Rv3131 ortholog has been reported during hypoxia^55^. Moreover, BCG likely up-regulates its Rv3131 ortholog upon internalisation by host cells after immunisation, as the DosR regulon is responsive to nitric oxide in host cell phagosomes^56^.

Despite the presence of the Rv3131 antigen in BCG, BCG-immunised mice exhibit a weaker response to Rv3131 than Rv3131-immunised mice in both the lung and the spleen. BCG, which is a live-attenuated *M. bovis* strain, does not actively grow in mice therefore it does not produce a sufficient level of Rv3131 *in vivo* for host immune system recognition. Thus, more susceptible mouse strains that develop human-like hypoxic granulomas in the lungs, such as C3Heb/FeJ, should be used as a murine vaccine model system to evaluate the protective efficacy of vaccine candidates derived from Rv3131 or the DosR regulon^57^,^58^. Given the BCG vaccination policy in South Korea, additional efficacy testing of Rv3131/GLA-SE as a booster vaccine and BCG overexpressing Rv3131 is ongoing in a pre-clinical model. In addition, further investigation of vaccine efficacy against non-Beijing Mtb strains should be completed to generate more general efficacy data and evaluate the efficacy of a BCG priming vaccination combined with Rv3131 subunit vaccination to determine whether Rv3131 subunit vaccination boosts BCG vaccine efficacy and whether Rv3131 could be a suitable antigen for TB subunit vaccine development. Testing Rv3131 subunit vaccine efficacy in an Mtb post-exposure model^36^ should be also considered, as Rv3131 is essentially a latency-associated Ag.

In summary, our present data demonstrate that DosR-encoded Rv3131, a novel target Ag for TB vaccine development, shows potential as an effective vaccine Ag by satisfying the following criteria: constitutive and stable expression in hyper-virulent Beijing Mtb; the ability to be recognised by the immune system during *in vivo* infection; the ability to induce Ag-specific, Th1-biased multifunctional T cells; and efficacy against hyper-virulent Mtb strains. Thus, Rv3131 may be an excellent candidate for use in a multi-antigenic Mtb subunit vaccine, especially considering the limited effectiveness of the BCG vaccine against the Beijing family.

## Methods

### Animals

All animal experiments were conducted in accordance with the Korean Food and Drug Administration (KFDA) guidelines and regulations. The experimental protocols were reviewed and approved by the Institutional Animal Care and Use Committee (Permit Number: 2015-0041) of Yonsei University Health System (Seoul, Korea). After approval of the study experiments, specific pathogen-free (SPF) female C57BL/6 mice at 6–7 weeks of age were purchased from Japan SLC, Inc. (Shizuoka, Japan) and maintained under barrier conditions in a BSL-3 facility at Avison Biomedical Research Center in Yonsei College of Medicine.

### Bacterial strains and preparation of bacterial RNA

Mtb H37Rv (ATCC 27294) was purchased from American Type Culture Collection (ATCC, Manassas, VA, USA), and the Mtb K strain was obtained from the strain collections at the Korean Institute of Tuberculosis (KIT, Osong, Chungchungbuk-do, Korea). BCG (Pasteur 1173P2) was kindly provided by the Pasteur Institute (Paris, France). The mycobacterial strains used in this study were cultured as described previously^59^. Total RNA was isolated from Mtb H37Rv and K strains grown to an optical density of OD_600_ = 0.5. For hypoxic condition, Mtb H37Rv and K strains grown to an optical density of OD_600_ = 0.2 were subjected to hypoxia as described by Wayne and Hayes^60^. The bacterial cell pellets were resuspended in Trizol (Invitrogen, CA, USA) and transferred to Lysing Matrix B tubes containing silica beads (MP Biomedicals, Germany). The tubes were agitated in a FastPrep homogeniser (Thermo Electron Corporation), and tubes were cooled on ice between each round of agitation. After centrifugation, RNA was prepared according to the recommendations of the manufacturer (Invitrogen). Then, DNase I (Ambion, Austin, TX, USA)-treated RNA was further purified using an RNeasy Mini Kit according to the manufacturer’s instructions (Qiagen).

### Microarray experiments and analysis

Mtb oligoarray slides were kindly provided by Dr. Stefan H. E. Kaufmann (Max Planck Institute for Infection Biology, Berlin, Germany). RNA labelling and array hybridisation were performed as described previously^59^. The hybridised slides were scanned using a GenePix 4000B microarray scanner (Axon Instruments, CA, USA), and spot intensities were identified and quantified with the TM4 Microarray Software Suite (http://www.tm4.org). The spot signal intensities were normalised in MIDAS using the LOWESS algorithm options and total array intensity. Four biological replicate arrays for exponential-growth condition and three biological replicates for hypoxic-culture condition were used for statistical analysis.

### Generation of BMDMs and *in vitro* infection

BMDMs were generated as previously described^61^. After 6 days, differentiated BMDMs were infected with Mtb H37Rv and K at an MOI of 10 for 24 h.

### Confirmation of *rv3131* expression by qRT-PCR

Total Mtb H37Rv and K RNA were extracted from exponential growth phase, under hypoxia and Mtb-infected BMDMs using Trizol as described above. Then, cDNA was synthesised using an RT-&GO Mastermix (MP Biomedicals, Germany) according to the supplier’s recommendations. After synthesis, differential gene expressions between Mtb H37Rv and K were measured using a qRT-PCR as previously described^62^. Briefly, qRT-PCR was performed on a StepOnePlus^TM^ Real-Time PCR system (Applied Biosystems, CA, USA) using SYBR^®^ Premix Ex Taq^TM^ II (Takara Bio Inc., Shiga, Japan). qRT-PCR was performed using the following primer sets; *rv3131* forward, 5′-GTGCCCTAGACCGAATGAAA-3′ and reverse, 5′-TAGCGCCCACTCCAAATG-3′, *dosR (rv3133c*) forward, 5′-AGACATCAAGGGAATGGAGTTG-3′ and reverse, 5′-TGGTCGGTAAGGCCTGATA-3′ and 16 S ribosomal RNA (rRNA) forward, 5′- CAACGCGAAGAACCTTACCT-3′ and reverse, 5′- CGGGACTTAACCCAACATCTC-3′. The fold change in gene expression between Mtb H37Rv and Mtb K was calculated using the ΔΔCt method, and 16 S rRNA was used for normalisation. Three biological replicates were used for statistical analysis.

### Expression and purification of recombinant Rv3131 protein

The *rv3131* gene was PCR amplified from Mtb H37Rv (ATCC 27294) genomic DNA with the following primer set: forward, 5′-CATATGACCGCAGCCGTTGA-3′ (Nde I restriction enzyme site is underlined) and reverse, 5′-AAGCTTGCACCGTTGTCGCA-3′ (Hind III restriction enzyme site is underlined). The amplified PCR product was inserted into the pET22b (+) vector (Novagen, WI, USA), and the *rv3131* sequence was analysed. Then, *rv3131*-inserted pET22b was transformed into *Escherichia coli (E. coli*) BL21. After cell lysis by sonication, recombinant Rv3131 was purified using a TALON Metal Affinity Resin (Clontech, CA, USA) and slight changes to a previously described method^63^. To eliminate endotoxin, Triton X-114 (Sigma, MO, USA) was added to the purified recombinant protein as previously described with slight modifications^64^. Finally, purified endotoxin-free recombinant protein was sterilised using a 0.22 μm filter and frozen at −70 °C until use. After 10% SDS-PAGE, the purity of the recombinant protein was confirmed by Coomassie brilliant blue staining and Western blot using an anti-histidine antibody (Santa Cruz Biotechnology, TX, USA). The Bradford protein assay was performed in accordance with the manufacturer’s instructions for quantification (Bio-Rad, CA, USA).

### Immunisation of mice

C57BL/6 mice were immunised through three intramuscular injections given three weeks apart. Each immunisation contained 5 μg of Rv3131 recombinant protein with 5 μg of GLA-SE (glucopyranosyl lipid adjuvant) formulated in an IDRI (Infectious Disease Research Institute) stable oil-in-water emulsion (SE). GLA-SE was kindly provided by IDRI (Seattle, WA, USA). For BCG immunisation, mice were injected subcutaneously with 2 × 10^5^ CFU of BCG Pasteur 1173P2. The control group of mice was immunised with GLA-SE only. Spleen and lung cells were collected and used to analyse immunogenicity at 4 weeks after the last immunisation.

### Mtb infection

Four weeks after the last immunisation, adjuvant control (GLA-SE) and vaccinated mice (BCG, Rv3131/GLA-SE) were infected aerogenically with Mtb K strain as previously described^63^. Briefly, mice were exposed to Mtb K for 60 min in the inhalation chamber of an airborne infection apparatus calibrated to deliver a predetermined dose (Glas-Col, Terre Haute, IN). To confirm the initial bacterial burden, four mice were euthanised one day later, and approximately 150 viable bacteria were delivered to the lungs of each mouse.

### Cytokine measurement

Single cells prepared from the spleens and lungs of Mtb-infected or immunised mice were stimulated with Rv3131 (5 μg/mL) or PPD (2 μg/mL) for 24 h at 37 °C. PPD was kindly provided by Dr. Brennan at Aeras (Rockville, MD, USA). The levels of secreted IFN-γ (eBioscience, San Diego, CA), IL-4 and IL-5 (BD Bioscience, San Diego, CA) in the culture supernatant were detected with a commercial ELISA kit according to the manufacturer’s instructions.

### Antibody titres in serum

Rv3131-specific IgG1 and IgG2c responses in serum were evaluated as previously described^63^. Briefly, 96-well plates were coated with 2 μg/mL Rv3131; a horseradish peroxidase (HRP)-conjugated antibody against IgG1 (BD Bioscience, San Diego, CA) or IgG2c (Southern Biotech, Birmingham, AL) was utilised as a secondary antibody. Optical densities (OD) were determined at 495 nm within 15 min of stopping the reaction.

### Analysis of T cell subpopulations

Single-cell suspensions of lung and spleen cells were prepared as previously described^65^. The single-cell suspensions were first blocked with anti-CD16/32 for 15 min at 4 °C. After the cell surface was stained with LIVE/DEAD^TM^ Fixable Aqua Dead cell kit (ThermoFisher Scientific, Waltham, MA, USA), Brilliant Violet 421 (BV421)-conjugated anti-CD3ε, peridinin chlorophyll (PerCP)-Cy5.5-conjugated anti-CD4, allophycocyanin (APC)-Cy7-conjugated anti-CD8 (BD Bioscience, San Diego, CA), phycoerythrin (PE)-conjugated anti-CD44, APC-conjugated anti-CD127 and fluorescein isothiocyanate (FITC)-conjugated anti-CD62L (eBioscience, San Diego, CA) antibodies for 30 min at 4 °C, the number of each type of memory T cell was analysed using a FACSVerse flow cytometer (BD Biosciences) and commercially available software (FlowJo).

### Intracellular cytokine staining

Single cell suspensions from immunised and infected mice (2 × 10^6^ cells) were stimulated with Rv3131 (5 μg/mL) or PPD (2 μg/mL) at 37 °C for 12 h in the presence of GolgiStop (BD Bioscience). Cells were first washed with PBS, blocked with an anti-CD16/32 blocking antibody at 4 °C for 15 min and then surface stained with fluorochrome-labelled antibodies against CD3ε, CD4, CD8 and CD62L as well as the LIVE/DEAD^TM^ Fixable Aqua Dead cell kit for 30 min at 4 °C. As negative controls, cells were surface stained with the proper isotype-matched immunoglobulin (Ig). Cells were washed, fixed and permeabilised with Cytofix/Cytoperm (BD Biosciences) for 30 min at 4 °C. Cells were washed twice with Perm/Wash (BD Biosciences) and then stained intracellularly with APC-conjugated anti-TNF-α, PE-conjugated anti-IFN-γ and PE-Cy7-conjugated anti-IL-2 (BD Biosciences) for 30 min at 4 °C. Cells were washed with Perm/Wash and then fixed with IC Fixation Buffer (eBioscience). After being resuspended in PBS, cells were analysed using a flow cytometer.

### Bacterial enumeration and histopathological analysis

Protection efficacy was determined by collecting the spleens and lungs from the infected mice at 4 and 10 weeks post-infection as previously described^51^. Briefly, the organ was homogenised, and serial dilutions were plated onto Middlebrook 7H11 agar plates (Becton Dickinson, Franklin Lakes, NJ, USA) supplemented with 10% OADC (Difco Laboratories), 2 μg/mL 2-thiophenecarboxylic acid hydrazide (Sigma-Aldrich, St. Louis, MO) and amphotericin B (Sigma-Aldrich) for bacterial growth. Bacterial colonies were counted after incubation at 37 °C for 3–4 weeks. The right-superior lobes of the lungs were collected for histopathology, preserved in 10% neutral buffered formalin overnight, embedded in paraffin, sectioned at 4–5 μm, and stained with H&E. Briefly, the stained right-superior lobes were examined for the severity of inflammation. The extent of inflammation in the lungs was analysed using the ImageJ program (National Institutes of Health, USA), as previously described^66^. For quantitative histopathological analysis of H&E staining, all slides were scanned using an Olympus BX43 microscope 4× objective (Olympus Optical Co., Tokyo, Japan), and a pathologist assessed and quantified histopathology in a blind manner. Each slide image was evaluated with the ToupTek Toup viewer program (Toup View Co., Zhejiang, China) to quantify the total size of the inflamed lesions in each mouse. The measured area is presented as mm^2^.

### Statistical analysis

All *in vitro* results are representative of at least three independent experiments with consistent results. The data in the graphs are presented as the mean ± standard deviation (SD). The data from the *in vivo* experiment are reported as the median with interquartile range (IQR). Comparisons between samples were made using an unpaired *t*-test or one-way ANOVA followed by Tukey’s multiple comparison test when data were normally distributed using Prism 5 (GraphPad Software version 5, San Diego, CA).

## Additional Information

**How to cite this article:** Kwon, K. W. *et al*. Novel vaccine potential of Rv3131, a DosR regulon-encoded putative nitroreductase, against hyper-virulent *Mycobacterium tuberculosis* strain K. *Sci. Rep.* **7**, 44151; doi: 10.1038/srep44151 (2017).

**Publisher's note:** Springer Nature remains neutral with regard to jurisdictional claims in published maps and institutional affiliations.

## Supplementary Material

Supplementary Information
