# Supplementary material for: Novel vaccine potential of Rv3131, a DosR regulon-encoded putative nitroreductase, against hyper-virulent Mycobacterium tuberculosis strain K
Source: Sci Rep. 2017 Mar 8;7:44151. doi: 10.1038/srep44151 (PMC5341159; doi:10.1038/srep44151)
Supplement: Supplementary Information [file srep44151-s1.doc]

**Novel vaccine potential of Rv3131, a DosR regulon-encoded putative nitroreductase, against hyper-virulent *Mycobacterium tuberculosis* strain K**

Kee Woong Kwon^1,2*^, Woo Sik Kim^1,2*^, Hongmin Kim^1,2^, Seung Jung Han^1,2^, Mi-Young Hahn^1^, Jong Seok Lee^3^, Ki Taek Nam^4^, Sang-Nae Cho^1,2^ and Sung Jae Shin^1,2^

^1^Department of Microbiology, ^2^Institute for Immunology and Immunological Diseases, Brain Korea 21 PLUS Project for Medical Science, Yonsei University College of Medicine, Seoul 03722, South Korea. ^3^Department of Microbiology Research, International Tuberculosis Research Center, Changwon 51755, South Korea. ^4^Severance Biomedical Science Institute, Brain Korea 21 PLUS Project for Medical Science, Yonsei University College of Medicine, Seoul 03722, South Korea.

^*^These authors contributed equally to this work.

Correspondence

Sung Jae Shin, Department of Microbiology and Institute of Immunology and Immunological Diseases, Yonsei University College of Medicine, Seoul 03722, South Korea. E-mail: sjshin@yuhs.ac, Tel.: (82) 2-2228-1813, Fax: (82) 2-2-392-9310

**Supplementary Information**

**Supplementary Table S1. List of highly up-regulated DosR regulon-related genes in Mtb K relative to Mtb H37Rv under different growth conditions.**

|  |  | Exponential phase | |  | Hypoxia | |  |  |
| --- | --- | --- | --- | --- | --- | --- | --- | --- |
|  |  |  | |  |  | |  |  |
| Locus | Gene | Fold change | *p*-value |  | Fold change | *p*-value |  | Function |
|  |  |  |  |  |  |  |  |  |
| Rv3131 |  | 5.62 | 0.003 |  | 2.69 | 0.025 |  | Conserved hypothetical protein |
| Rv3127 |  | 5.37 | 0.0006 |  | - | - |  | Conserved hypothetical protein |
| Rv3130c | *tgs1* | 5.33 | 0.0025 |  | - | - |  | Conserved hypothetical protein |
| Rv2007c | *fdxA* | 5.17 | 0.0104 |  | - | - |  | Ferredoxin |
| Rv3133c | *dosR* | 4.2 | 0.0018 |  | 2.05 | 0.054 |  | Two-component transcriptional regulator |
| Rv2626c |  | 3.49 | 0.0003 |  | 2.06 | 0.0097 |  | Conserved hypothetical protein |
| Rv1738 |  | 4.27 | 0.0333 |  | - | - |  | Conserved hypothetical protein |
| Rv2031c | *hspX* | 3.96 | 0.0053 |  | - | - |  | Heat shock protein |
| Rv2628 |  | 3.87 | 0.0359 |  | 2.64 | 0.0093 |  | Hypothetical protein |
| Rv2030c |  | 3.45 | 0.0057 |  | 2.32 | 0.0392 |  | Conserved hypothetical protein |
| Rv3132c | *dosS* | 2.97 | 0.0123 |  | - | - |  | Two-component sensor histidine kinase |
| Rv3134c | *phyA* | 2.91 | 0.0172 |  | - | - |  | Conserved hypothetical protein |
| Rv2623 |  | 2.73 | 0.0235 |  | - | - |  | Conserved hypothetical protein |
| Rv3126c |  | 2.29 | 0.0096 |  | - | - |  | Hypothetical protein |
| Rv1733c |  | 2.65 | 0.0181 |  | - | - |  | Conserved membrane protein |
| Rv1813c |  | 2.64 | 0.0348 |  | 2.39 | 0.032 |  | Conserved hypothetical protein |
| Rv2029c | *pfkB* | 2.5 | 0.0389 |  | 2.85 | 0.0479 |  | Phosphofructokinase |
| Rv1737c | *narK2* | 2.1 | 0.038 |  | 4.52 | 0.0122 |  | Nitrate/nitrite transpoter |
| Rv0080 |  | 2.09 | 0.0433 |  | 1.9 | 0.0475 |  | Conserved hypothetical protein |

The expression levels of the DosR regulon-related gene transcripts in the Mtb H37Rv and K strains both exponentially grown and under hypoxia were investigated using a microarray. Among the up-regulated DosR regulon-related genes under exponential growth condition, only those with an average expression level 2-fold higher in Mtb K relative to Mtb H37Rv are displayed, and a fold changes of these up-regulated genes in Mtb K are further analysed under hypoxia. Four biological replicate arrays for exponential-growth condition and three biological replicates for hypoxic-culture condition were used for statistical analysis. -: No differential expression between Mtb H37Rv and K strains.

**Supplementary figures**

**Figure S1. Production of Th2-related cytokines with the ex vivo stimulation of Rv3131 after final immunisation.**

**
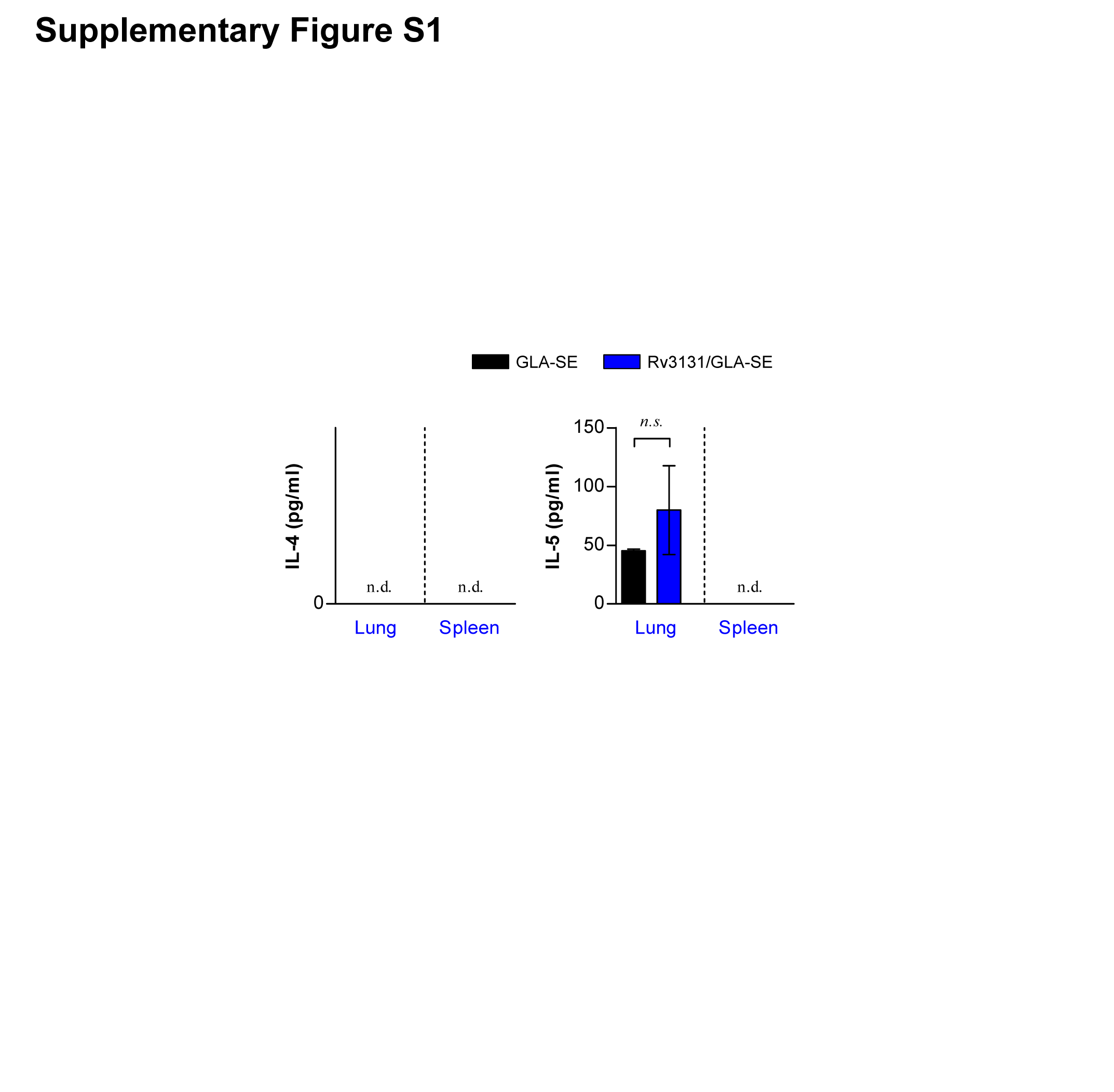
**

The level of IL-4 and IL-5 secreted by lung and spleen cells from each fully immunised group in response to Rv3131 (5 μg/mL) stimulation was determined with ELISA. The significance of the differences compared to GLA-SE-immunised mice was determined using an unpaired *t*-test. A *p* value < 0.05 was considered statistically significant. *n.s.*: non-significant; n.d.: not detected.

**Figure S2. Gating strategy for the flow cytometry analysis of effector/memory T cell subpopulations employed in Figure 2.**


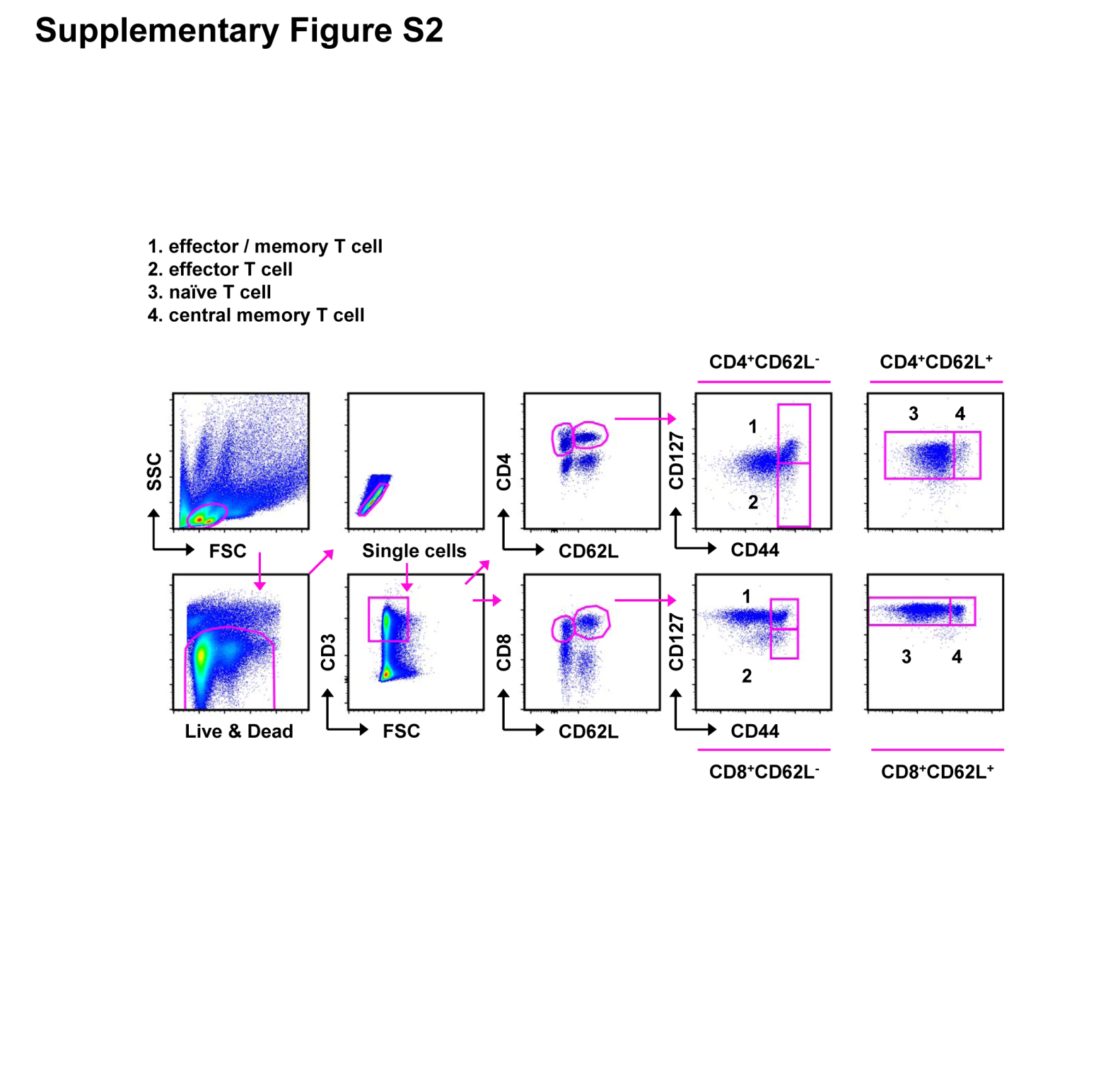


Four weeks after the final immunisation with Rv3131/GLA-SE, the lung and spleen cells from each immunised group (5 mice per group) were prepared as described in the Methods. Forward scatter (FSC) and side scatter (SSC) were used to establish a gate around lymphocytes; then, a gated live cell population was further drawn around single cells to exclude aggregated cells on the basis of FSC-area and FSC-height values. For analysis of the memory T cell subpopulation, CD3^+^CD4^+^ and CD3^+^CD8^+^ T cells were further gated into effector/memory T cells (CD62L^-^CD44^+^CD127^+^), effector T cells (CD62L^-^CD44^+^CD127^-^), naïve T cells (CD62L^+^CD44^-^CD127^+^) and central memory T cells (CD62L^+^CD44^+^CD127^+^). Finally, data were analysed using FlowJo software.

**Figure S3. Induction of multifunctional T cells in response to PPD in each group of mice after challenge with Mtb K.**


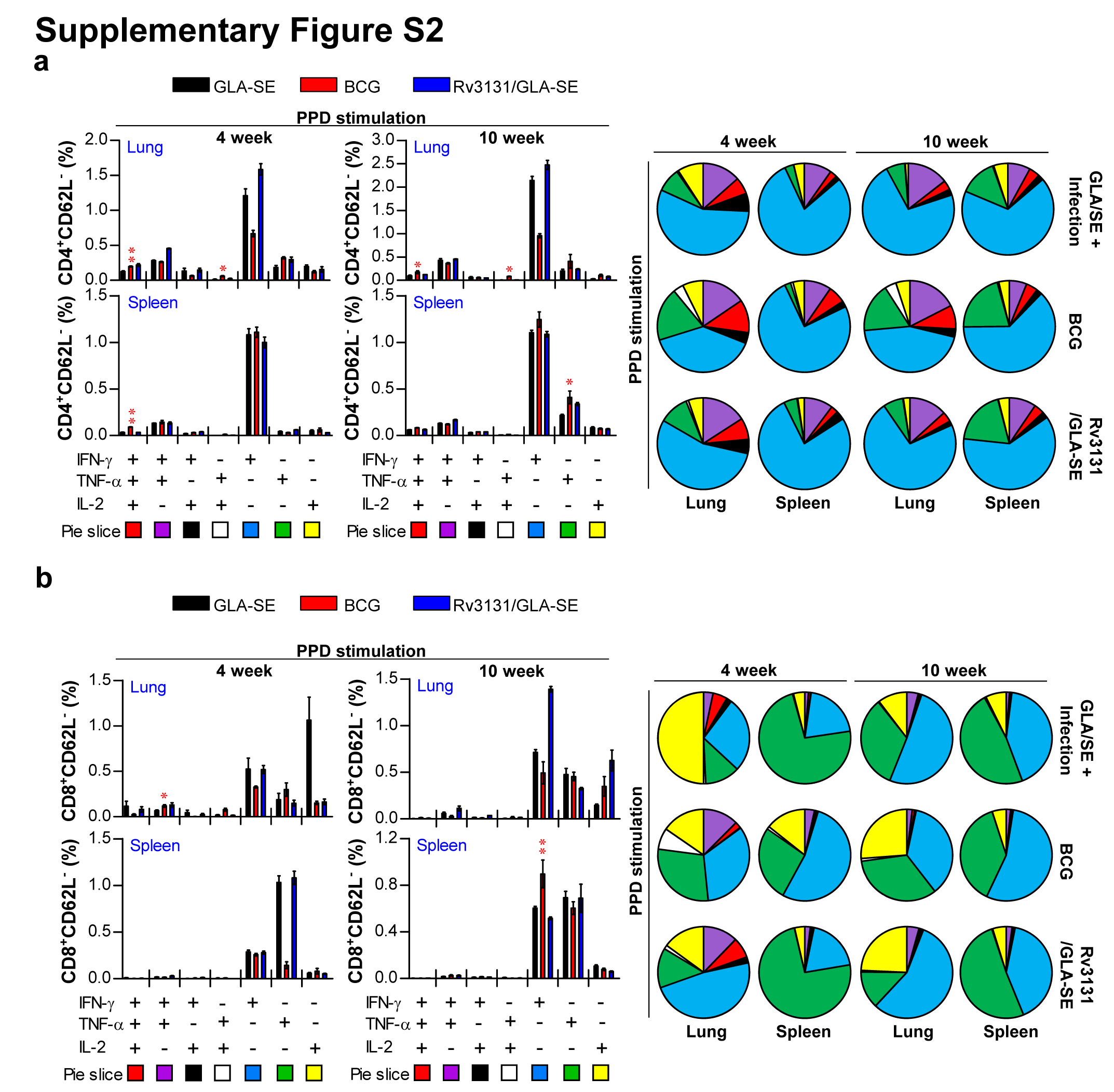


The mice in each group were sacrificed at 4 or 10 weeks post-infection, and their lung and spleen cells were stimulated with PPD (2 μg/mL) at 37 ˚C for 12 h in the presence of GolgiStop. The gating strategy for flow cytometry analysis shown in Figure 3 was employed. Upon stimulation, the percentages of Ag-specific, multifunctional CD4^+^CD62L^-^ (a) and CD8^+^CD62L^-^ (b) T cells producing TNF-α, IFN-γ and/or IL-2 in each immunised group were evaluated using flow cytometry. The mean frequencies of cells producing effector cytokines are shown as pie charts. The results are expressed as the mean ± SD from 6 mice in each group. The significance of the differences was determined using an unpaired *t*-test. A *p* value < 0.05 was considered statistically significant. **p* < 0.05, ***p* < 0.01, and ****p* < 0.001 compared to the GLA-SE-alone group.
